# Point-of-Care Testing Using a Neuropsychology Pocketcard Set: A Preliminary Validation Study

**DOI:** 10.3390/brainsci12060694

**Published:** 2022-05-27

**Authors:** Emily Bellartz, Milena Pertz, Johannes Jungilligens, Ilka Kleffner, Jörg Wellmer, Uwe Schlegel, Patrizia Thoma, Stoyan Popkirov

**Affiliations:** 1 Department of Neurology, University Hospital Knappschaftskrankenhaus Bochum, Ruhr University Bochum, 44892 Bochum, Germany; emily.bellartz@ruhr-uni-bochum.de (E.B.); milena.pertz@kk-bochum.de (M.P.); johannes.jungilligens@kk-bochum.de (J.J.); ilka.kleffner@kk-bochum.de (I.K.); uwe.schlegel@kk-bochum.de (U.S.); 2 Neuropsychological Therapy Centre (NTC)/Clinical Neuropsychology, Faculty of Psychology, Ruhr University Bochum, 44801 Bochum, Germany; patrizia.thoma@rub.de; 3 Ruhr-Epileptology, Department of Neurology, University Hospital Knappschaftskrankenhaus Bochum, Ruhr University Bochum, 44892 Bochum, Germany; joerg.wellmer@kk-bochum.de

**Keywords:** neuropsychology, pocketcard, aphasia, neglect, executive functions, stroke, epilepsy

## Abstract

Neurocognitive screening instruments usually require printed sheets and additional accessories, and can be unsuitable for low-threshold use during ward rounds or emergency workup, especially in patients with motor impairments. Here, we test the utility of a newly developed neuropsychology pocketcard set for point-of-care testing. For aphasia and neglect assessment, modified versions of the Language Screening Test and the Bells Test were validated on 63 and 60 acute stroke unit patients, respectively, against expert clinical evaluation and the original pen-and-paper Bells Test. The pocketcard aphasia test achieved an excellent area under the curve (AUC) of 0.94 (95% CI: 0.88–1, *p* < 0.001). Using an optimal cut-off of ≥2 mistakes, sensitivity was 91% and specificity was 81%. The pocketcard Bells Task, measured against the clinical neglect diagnosis, achieved higher sensitivity (89%) and specificity (88%) than the original paper-based instrument (78% and 75%, respectively). Separately, executive function tests (modified versions of the Trail Making Test [TMT] A and B, custom Stroop color naming task, vigilance ‘A’ Montreal Cognitive Assessment item) were validated on 44 inpatients with epilepsy against the EpiTrack^®^ test battery. Pocketcard TMT performance was significantly correlated with the original EpiTrack^®^ versions (A: *r* = 0.64, *p* < 0.001; B: *r* = 0.75, *p* < 0.001). AUCs for the custom Stroop task, TMT A and TMT B for discriminating between normal and pathological EpiTrack^®^ scores were acceptable, excellent and outstanding, respectively. Quick point-of-care testing using a pocketcard set is feasible and yields diagnostically valid information.

## 1. Introduction

The neurological exam is an invaluable clinical instrument for diagnosing disorders of the nervous system. Bedside testing of vigilance, cranial nerve function and the motor system have been honed by generations of neurologists and validated by a myriad of studies. Widely used clinical scales such as the National Institute of Health Stroke Scale (NIHSS) can effectively guide treatment even in emergency situations when quick assessments and a timely diagnosis are essential [1]. Higher-order cognitive deficits, although common across neurological conditions, are often neglected in acute clinical practice, and neurologists mostly rely on subjective impressions rather than structured bedside tests.

Surveys have shown that a large majority of speech and language therapists (SLT) do not use standardized assessments to detect aphasia [2], and similarly, most occupational therapists providing inpatient stroke care do not use standardized assessments for neglect [3]. Acute stroke physicians are likely to overlook neuropsychological deficits—and thus potential rehabilitation needs—when systematic screening is not implemented [4,5]. Aphasia and neglect assessment in acute stroke patients is often performed using the NIHSS and similar scales, which are, however, not validated for these domains [1,6]. NIHSS aphasia assessment (item 9) misses 28% of aphasias detected by the two-minute Language Screening Test (LAST) [7]. With a sensitivity of 32%, the NIHSS neglect assessment (item 11) detects only one in three patients with neglect compared with comprehensive cognitive testing [8].

Impairments of executive function are highly prevalent among neurological patients [9]. Although neuropsychological workup is established in most neurological departments, bedside examinations of executive function are rarely performed outside of formal testing. Screening instruments such as the Montreal Cognitive Assessment (MoCA) and the Mini Mental State Exam include executive function subtests, but their overall scores are only validated to detect clinical syndromes such as mild cognitive impairment and dementia, without allowing further differentiation. However, isolated executive dysfunction can provide valuable diagnostic clues in a wide range of disorders. For example, in epileptology, new-onset impairments in executive function are a sensitive indicator of anticonvulsive medication side effects, for which specific test batteries (such as the EpiTrack^®^ used in this study) have been validated [10].

Although a range of pen-and-paper and app-based cognitive screening instruments exist, they are not widely implemented in emergency workup or on routine ward rounds. The reasons for this are manifold, but lack of low-threshold access to equipment (testing sheets, mobile devices, etc.), and impaired mobility of patients are likely obstacles. Especially in acute stroke patients, impaired sitting and writing abilities are common. Considering the busy reality of emergency and inpatient neurology practice, and the everyday obstacles to implementing neuropsychology screening instruments (patient mobility, hygienic considerations, equipment availability, time, cost, etc.), a low-threshold multimodal bedside testing instrument would be useful.

Inspired by the ubiquitous use of pocket-sized eye charts for bedside assessment of visual acuity among neurologists, a pocketcard set with a range of neuropsychological tests was developed. The set includes a range of short versions of established neuropsychological tests. Just as eye chart bedside testing can aid diagnostic decision-making, but would not be used for prescribing eyeglasses, this neuropsychology pocketcard set is not intended to replace formal testing, but rather to support the neurological exam at the bedside. It is meant as an intermediate step of neurological examination between the clinician’s subjective impression and formal neuropsychological testing.

To test the utility of this pocketcard set, a clinical study regarding feasibility and preliminary validation on a prospective sample of inpatients in a stroke unit or on an epileptology ward was undertaken.

## 2. Materials and Methods

### 2.1. Patients

Patients were recruited over a period of five months at the Department of Neurology of the University Hospital Knappschaftskrankenhaus Bochum, which has an embedded quaternary epilepsy center (Ruhr-Epileptology). Testing was split between two patient populations (stroke and epilepsy), as this would allow the use of routinely acquired reference assessments as well as ensure sufficient prevalence of respective deficits (aphasia/neglect and executive dysfunction, respectively). For aphasia and neglect testing a prospectively recruited convenience sample of patients treated in the stroke unit with a diagnosis of ischemic or hemorrhagic stroke (including transient ischemic attack) was recruited. For executive function testing a convenience sample of inpatients with an epilepsy diagnosis was recruited. All patients had to be at least 18 years of age and have sufficient German language proficiency to provide consent and understand basic instructions. Exclusion criteria were significant impairments in wakefulness, severe vision impairment (blindness or near-blindness), major hearing impairment, cardiopulmonary instability, severe infection, known major cognitive impairment due to encephalopathy or dementia and an acute postictal state. Individual eligibility and ability to provide consent was affirmed by a treating physician for each case; screening and recruitment was performed by the testing psychologist (E.B.). As testing was not available every day and patient turnover in the stroke unit is quick, not all eligible patients were screened, and unscreened or ineligible patients were not recorded or characterized. Most of the epilepsy patients were scheduled for regular routine diagnostic neuropsychological testing.

Since this study aimed at assessing criterion validity of the pocketcard tests in relation to established diagnostic procedures, additional clinical parameters relating to disease etiology (e.g., ischemic or hemorrhagic stroke, form of epilepsy, antiseizure medication), patients characteristics (e.g., age and sex) and clinical presentation (e.g., type and severity of aphasia according to speech and language therapist evaluation) did not affect recruitment and were not further evaluated. Indeed, the variability of neuropsychological impairment inherent in using convenience sampling irrespective of etiology, medication, age and other determinants was welcomed, as it allowed for a more representative correlation analyses between experimental task performance and the criteria it was validated against.

The study was approved by the Ethics Committee of the Medical Faculty of the Ruhr University Bochum (Reg.-Number: 21-7151).

### 2.2. Testing Procedure

All forms of aphasia and neglect testing were completed at the bedside in the stroke unit. Testing was conducted after hyperacute stroke workup and treatment was completed. While care was taken to minimize distractions (e.g., turn off radio, ask relatives to wait outside), the setting was as distractible as a two-bed intermediate care monitoring room would normally be.

Executive function testing using the pocketcard set was performed at the bedside in regular hospital rooms. Here also, distractions were minimized as far as possible.

The EpiTrack^®^ testing battery (description below) was completed directly after the bedside pocketcard testing, but in a dedicated neuropsychology examination room (part of routine neuropsychology testing for epilepsy patients). All neuropsychological testing (pocketcard and paper based) was conducted by the same psychologist (E.B.).

### 2.3. Neuropsychology Pocketcard Set

**Figure 1:**
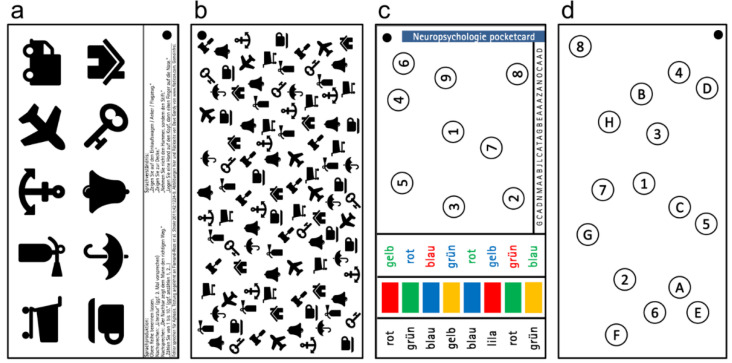
Neuropsychology pocketcard set. The four pages of the pocketcard set used in this study, including tests for aphasia (**a**), neglect (**b**) and executive functions (**c**,**d**).

The neuropsychology pocketcard set consisted of four laminated 9.5 cm × 8.7 cm pages (Figure 1) that could comfortably fit in the pocket of a standard white coat and could be cleaned with regular surface disinfectants.

Page 1 (Figure 1a) is used for aphasia screening and consists of 10 black-and-white visual stimuli alongside instructions (pictograms were retrieved from www.flaticon.com, accessed on 20 July 2020, and used under creative commons attribution license, author: Dave Gandy). The testing procedure was a modified version of the original and the German version of the Language Screening Test (LAST) using visual stimuli without shading, color or detail [11,12]. When asked to name or point at the pictures, patients were presented the card within reading/reaching distance. When patients were unable to sit up for testing the card was held at eyelevel above them. For one of the instruction comprehension tasks (“Please do not take the tendon hammer, but rather take the pen”) the examiner would present both utensils in front of the patient in the palm of their (the presenters) hand. It was assumed that neurologists would have easy access to these objects on most occasions. Overall, the patient received one point for each correct answer for a maximum total score of 15 points.

Page 2 (Figure 1b) was a modified version of the Bells Test [13], used to identify spatial neglect symptoms. The card consists of an assortment of 102 diffusely distributed objects (9 different objects from the language testing card), including 13 identical bells. The pocketcard was held centrally in front of the patient’s eyes at a distance of about 20 cm, irrespective of the position the patient is in (supine, sitting, turned slightly to the side). The bell at the top middle of the card was shown as an example of the target stimulus. The patient was then asked to point at all the remaining bells they can find. There was a total of 12 remaining bells evenly distributed to both sides of the card. Performance was scored by determining the difference in the number of identified items between both sides (asymmetry index).

Page 3 and 4 (Figure 1c,d) included modified versions of the Trail Making Test (TMT) A and B, used to assess executive functions. The card was held in front of the patient, who was then asked to point at the numbers in ascending order as quickly as possible starting at 1 (TMT A), or to point in alternation at the numbers and letters in order (TMT B). Errors were corrected immediately by the examiner (e.g., “You were at 4. Which is the next letter? Please continue from there.”). The time to task completion was measured using a stopwatch during the validation study. The number of errors was also recorded. A custom modified Stroop color naming task inspired by the original [14] was also included. Patients were asked to first read out the eight color words in the first column; then to name the eight colors shown in the second column; once reading and color identification ability had been ascertained, the patients were asked to name the font color of the eight color words in the third column. Errors in this subtask that were not self-corrected were counted. A slightly modified version of the vigilance item from the MoCA test was also included on page 4 [15]. Here, a series of pseudo-randomized letters were individually read to the patient (1/s), who was instructed to tap with the hand each time they hear the letter “A”.

### 2.4. Clinical Data

For stroke patients the following data was extracted from routine clinical documentation: age, sex, diagnosis, presence of aphasia and/or neglect according to last physician examination, presence of aphasia according to last speech and language therapist evaluation.

To assess criterion validity of the aphasia testing, results were measured against binary aphasia evaluation (aphasia vs. no aphasia) by a trained SLT with long-standing experience with acute stroke patients. SLT evaluation is routinely performed when any language, speech or swallowing impairment is observed or suspected, i.e., in most but not all stroke unit admissions. As this was a feasibility study aiming only at preliminary validation, SLT evaluation was not standardized or performed using comprehensive test batteries, but carried out as usual.

### 2.5. Additional Testing

Stroke unit patients tested for neglect using the pocketcard were subsequently evaluated using the original Bells Test using standard testing procedures [13]. In addition, confrontational neglect testing was performed by the examiner for visual and tactile extinction as defined for the NIHSS [1]: the patient was first asked if they noticed finger wiggling (for visual testing) or light touch (for tactile testing) on the left and then right side (of visual field/lower arm); then, upon bilateral stimulation, the patient was asked on which side they perceived movement/touch. Extinction was noted when both sides were perceived separately but only one side was noticed upon simultaneous stimulation.

Standard neuropsychology testing for epilepsy patients was performed in a dedicated examination room using the EpiTrack^®^ testing battery [10]. The EpiTrack^®^ was originally developed to assess attention and executive functions in epilepsy patients, and includes an interference/response inhibition test, TMT A and B, a maze test, verbal fluency and digit span backwards. One overall, age-corrected score is computed, which is used to rate the performance as unimpaired, borderline or impaired (score ≤ 28 points). Psychometric qualities are good, with a Cronbach’s alpha of 0.75 and a test–retest reliability of 0.79.

### 2.6. Statistical Analysis

Diagnostic utility was assessed by calculating receiver operating characteristic (ROC) curves, the area under the curve (AUC) and determining optimal cut-off values for optimal sensitivity/specificity values by finding the highest Youden index (sensitivity + specificity—1). An AUC of 0.7 to 0.8 was considered acceptable, 0.8 to 0.9 was considered excellent and more than 0.9 was considered outstanding [16]. Four-field tables were used to calculate sensitivity and specificity of extinction testing, 95%-confidence intervals (95% CI) were calculated and significance level was set at *p* < 0.05.

## 3. Results

### 3.1. Aphasia and Neglect Testing on Stroke Unit Patients

A total of 78 stroke unit patients (mean age 66.4 years (range: 27–90)) were eligible, able to consent, and willing to participate. One patient was later excluded when the severity of a pre-existing dementia became apparent and consent had to be annulled, leaving a total of 77 stroke unit patients tested with the pocketcard set for aphasia and neglect. One patient did not complete aphasia testing and five patients did not complete neglect screening for various reasons (problems with fully comprehending instructions and/or severely restricted mobility). This rate (92%) of successful testing confirmed the feasibility of using a pocketcard set for point-of-care neuropsychology testing on acutely ill patients in a stroke unit.

Overall, 63 of tested stroke unit patients were also evaluated by an SLT (mean age 67.5 years, age range 27 to 90 years, 22 female) with mostly mild stroke severity (mean NIHSS score on day of testing: 2.95; median: 2; interquartile range: 1–4). In nine cases (14%) some degree of aphasia was noted. Using the original cut-off of ≥1 point (mistake) and measured against this evaluation, the pocketcard aphasia test achieved a sensitivity of 100% and a specificity of 65.4%. An ROC was calculated and yielded an AUC of 0.94 (95% CI: 0.88–1, *p* < 0.001). With a Youden index of 0.63 the optimal cut-off was ≥2 mistakes indicating aphasia, which resulted in a sensitivity of 90.9% and a specificity of 80.8%.

Of the 72 patients who underwent neglect testing, 6 patients (8%) had a neglect to the left side and 4 (6%) to the right side according to the overall clinical judgement recorded by the treating physician team. Of the remaining 62 patients, the absence of neglect was documented in 51, and in 11 patients no documentation regarding presence or absence of neglect near the time point of pocketcard testing could be retrieved. For validity testing, only data from the 61 patients with clear documentation regarding neglect were used (mean age 65.5 y; age range: 36–87; 22 female; mean NIHSS score on day of testing: 3; median: 2; interquartile range 0–4). Furthermore, some elements of the neglect testing were not performed or evaluable in individual patients, so the number of tested individuals will be provided for each of the following calculations.

Spearman rank correlation analysis showed a highly significant correlation of moderate strength between the number of identified items on the original pen-and-paper Bells Test and the modified pocketcard version (*n* = 70; left side: *r* = 0.58, *p* < 0.001; right side: *r* = 0.49, *p* < 0.001).

The ROC of the original Bells Test revealed an excellent AUC of 0.80 (95% CI: 0.62–0.98; *p* = 0.004) with an optimal cut-off of ≥5 more missed items on one side compared with the other side (Youden index 0.59), yielding a sensitivity of 67% and a specificity of 92%. However, the literature suggests using a lower cut-off of ≥3 [17], which will be applied for further analysis, and which in our sample corresponded to a Youden index of 0.52 for a sensitivity of 77.8% and a specificity of 74.5%.

The ROC of the pocketcard Bells Test revealed an outstanding AUC of 0.96, (95% CI: 0.90–1.00; *p* < 0.001) with maximal Youden index of 0.77 at a cut-off of ≥2 more missed items on one side compared with the other side, corresponding to a sensitivity of 89% and a specificity of 88%.

Table 1 shows the sensitivity and specificity regarding overall clinical judgement of the neglect assessments used in this study compared with the overall clinical judgement by the treating stroke unit team.

### 3.2. Executive Functions Testing on Epilepsy Patients

A total of 44 epilepsy patients (mean age 40.7 years, age range 19–74 years, 29 female) were eligible and able to consent, as determined by treating physicians, and gave written consent to participate. Two patients did not complete the entire EpiTrack^®^ (one did not complete TMT B and two did not complete the digits backwards task). Using the established cut-off of 28 points, 9 (21.4%) of 42 fully tested epilepsy patients had a pathological EpiTrack^®^ performance and were thus classified as having an impairment in executive functions. As explained in the methods, the reason for impairment (brain pathology, antiseizure medication, etc.) was not further characterized.

Looking at individual task performance, the results of the modified pen-less trail making tests of the pocketcard (duration to complete measured in seconds) correlated significantly with the performance of the respective pen-and-paper tasks in the EpiTrack^®^ battery across all 44 patients (Table 2). For the TMT-A, the Spearman rank correlation coefficient was *r* = 0.64 (*p* < 0.001); for TMT-B it was *r* = 0.75 (*p* < 0.001). Derived measures that can be used to differentiate between general slowing and reduced mental flexibility (TMT B-A and TMT B/A) were also significantly correlated between pen-and-paper and pocketcard tests (*r*_B-A_ = 0.63, *p* < 0.001; *r*_B/A_ = 0.38, *p* = 0.013).

As screening tests regarding pathological impairment of executive function detected through the validated EpiTrack^®^ test battery, the four pocketcard tests were assessed for their diagnostic precision by calculating the AUC for the respective ROC (Table 2).

## 4. Discussion

Using a newly developed pocketcard set as a bedside screening instrument for aphasia, neglect and executive dysfunction proved feasible in acutely ill neurological patients. Preliminary validation revealed acceptable to outstanding diagnostic performance of all but one test (MOCA vigilance item) in comparison with expert clinical judgement or established tests.

Bedside testing of stroke unit patients showed that a pocketcard implementation of a modified LAST using a slightly higher cut-off of ≥2 mistakes detected SLT-confirmed aphasia with a sensitivity of 90.9% and a specificity of 80.8%. This performance was not as good as that of the original LAST (sensitivity: 98%; specificity: 100%) [11]. In addition to the modifications made to the test (especially the change of visual stimuli), our study population and setting were different; while our study was undertaken with acutely ill patients monitored in two-bed stroke unit rooms, the original external validation study was performed on stabilized “chronic” stroke patients outside the stroke unit, who were able to complete the entire Boston Diagnostic Aphasia Evaluation. The reduced diagnostic precision and the higher optimal cut-off in our study suggest that patients might have made errors for reasons other than aphasia, such as attentional deficits, external distractions, unavailability of hearing/visual aids, dysarthria and other. Furthermore, the validation criterion of professional but non-standardized SLT assessment might have missed subtle deficits that the pocketcard test picked up, leading to false false-positives. With a full rate of test completion and a sensitivity above 90%, the modified pocketcard version of the LAST could be fully implemented in acute stroke unit care and is suitable for quick bedside screening for aphasia when a printed version of the original LAST or other testing materials are not on hand.

In comparison with the original Bells Task, the pocketcard version achieved higher sensitivity and specificity regarding neglect in acute stroke patients as evaluated by the clinical neurology team. This difference is likely due to problems with pen-and-paper-based testing in this setting. Sensorimotor impairments and a variety of monitoring cables and catheters possibly hindered the completion of the pen-and-paper Bells Test. Alternatively, as our comparison criterion for this preliminary validation was overall clinical judgement of the treating neurologists, again, false false-positives are conceivable. By comparison, visual and tactile extinction testing, which is the neglect test of choice for the NIHSS, had a relatively low sensitivity, in line with previous studies [8]. Overall, these results strongly support the use of this pocket-sized gesture-based neglect assessment instrument for bedside use.

Pocketcard TMT performance was significantly correlated with the original versions within EpiTrack^®^ (A: *r* = 0.64, *p* < 0.001; B: *r* = 0.75, *p* < 0.001), which provides preliminary validation for a gesture-based test format compared with the extensively validated original form of line drawing. Measuring the time taken to complete TMT A and TMT B yielded excellent and outstanding AUCs, respectively, for identifying patients with executive dysfunction according to the EpiTrack^®^. Despite the ubiquity of stopwatch apps on smartphones, this form of testing entails some of the obstacles to routine use that the pocketcard aims to avoid. Future studies should probe whether a pathologically slow performance on the trail making tasks can be intuited by examiners watching the patient point at the numbers and letters in order. Alternatively (or in addition), observing an error on TMT B suggests executive dysfunction with a sensitivity of 78% and a specificity of 73%. A second error raises the specificity to 91%. The very brief Stroop task similarly had an acceptable AUC suggesting usability in clinical practice, while the MoCA vigilance task on its own did not appear to be diagnostically useful in our sample. Taken together these results suggest that quick point-of-care screening for executive dysfunction using the pocketcard is feasible and useful in acute neurology care.

It is essential to emphasize the preliminary nature of this validation study and to acknowledge its limitations. For all neuropsychological domains tested, more comprehensive and thoroughly validated tests and test batteries exist, such as the Aachener Aphasie Test for aphasia, the Behavioral Inattention Test for neglect or the Behavioral Assessment of Dysexecutive Syndrome for executive functions [18,19,20]. As the aim of this study, besides preliminary validation, was to test the feasibility of implementing bedside testing in acutely ill stroke unit patients, comprehensive reference assessments that take 40–60 min to complete were deemed inappropriate. The dynamic nature of acute cerebrovascular disorders means that neuropsychological deficits can change rapidly. On the stroke unit SLT and neurological assessments are documented daily and several times daily, respectively. The assessment nearest to the time point of pocketcard testing was retrieved from medical records, but the potential for between-testing variation still introduces an important risk of error. Convenience sampling means that patients with more severe forms of communication deficits (aphasia, dysarthria, psychomotor retardation, etc.) could not consent and were not included. However, in severely impaired individuals deficits would likely be obvious and screening procedures thus unnecessary. These considerations, together with the fact that clinical characteristics were not further analyzed, limit the generalizability of these preliminary validation results. Lastly, the current study does not provide sufficient validation data to allow for direct comparisons with alternative pocket-sized screening instruments [21].

Overall, the positive results of this preliminary validation warrant further testing. Specifically, the pocketcard tests should be performed at the same sitting as comprehensive gold standard SLT/neuropsychological assessment in a more diverse neurological clinical population. The utility of executive function testing to detect delirium in neurological, geriatric and intensive care unit patients should be examined, as this would be a potential field of application. This preliminary validation suggests that the pocketcard set can be clinically useful in neurological populations to complement the neurological bedside exam when more comprehensive validated screening procedures are not available. Positive screening results should prompt formal neuropsychology testing and should inform treatment and rehabilitation strategies.

## Figures and Tables

**Table 1:** Neglect testing procedures compared with overall clinical judgement by the treating stroke unit team.

Test	Sample Size	Sensitivity	Specificity
Original Bells Test *	60	77.8%	74.5%
Modified pocketcard Bells Test	60	88.9%	88.2%
Tactile extinction	60	33.3%	95.0%
Visual extinction	61	60.0%	92.2%
Combined extinction testing	61	60.0%	92.2%

* using literature cut-off.

**Table 2:** Screening test performance regarding pathological impairment of executive function detected through EpiTrack^®^ (*n* = 42).

Pocketcard Test	AUC (95% CI; *p*)	Youden Index	Optimal Cut-Off	Sensitivity	Specificity
TMT A (errors)	0.56 (0.33–0.78; 0.613)	0.11	≥1 error	11%	100%
TMT B (errors)	0.79 (0.61–0.97; 0.008)	0.50	≥1 error	78%	73%
TMT A (duration)	0.81 (0.63–0.98; 0.005)	0.57	≥7 s	67%	90%
TMT B (duration)	0.92 (0.81–1; <0.001)	0.75	≥48 s	78%	97%
Stroop Test	0.79 (0.61–0.97; 0.008)	0.63	≥1 error *	78%	85%
Vigilance item	0.67 (0.45–0.88; 0.13)	0.31	≥1 error	56%	76%

* not self-corrected.

## Data Availability

Study data available upon reasonable request.
